# Characterization of the complete chloroplast genome of the endangered Chinese species *Cymbidium lowianum (Rchb.f.) Rchb.f.*

**DOI:** 10.1080/23802359.2020.1815603

**Published:** 2020-09-04

**Authors:** Longjie Cheng, Yuying Wang, Yiran Zhao, Yefang Li, Fengmei He, Zhilin Li

**Affiliations:** College of Horticulture and Landscape, Yunnan Agricultural University, Kunming, China

**Keywords:** *Cymbidium lowianum*, chloroplast genome, endangered species, phylogenetic analysis

## Abstract

*Cymbidium lowianum (Rchb.f.) Rchb.f.* is a Class I endangered species in China with important ornamental, economic, and breeding value, especially wild *C. lowianum.* This study used Illumina high-throughput sequencing technologies to sequence and analyze the complete chloroplast genome of *C. lowianum*. The genome features of *C. lowianum* and the phylogenetic relationships among Orchidaceae species were reported and established. The complete chloroplast genome is 155,447 bp long, consisting of a pair of inverse duplication regions that are 26,710 bp long, a large single-copy region of 84,184 bp, and a small single-copy region of 17,843 bp. The entire genome contains 74 mRNA genes, 30 tRNA genes, and 4 rRNA genes. The phylogenetic tree of 24 Orchidaceae species revealed *Cymbidium lowianum* is more closely related to *Cymbidium erythraeum*.

*Cymbidium lowianum (Rchb.f.) Rchb.f.* (Orchidaceae) is a shrub that is an endemic species in Yunnan Province, China. Wild *C. lowianum* grows on trees in forests and cliffs along valleys at elevations of 1300–1900 m (Liu et al. 2009). It has been listed as a Class I protected plant in the China Biodiversity Red List and in the China Rare and Endangered Plants List (http://www.iplant.cn/rep/protlist). *Cymbidium lowianum* is an excellent garden plant with ornamental flowers that have striking, lip with a deep red V-shaped formation. The length of the inflorescence is 60–80 cm with 10–20 or more flowers, and the flowering period ranges from April to May (Li et al. 2010). Outside of the flowering season, *C. lowianum* is appreciated for its gracefully shaped evergreen foliage. Due to its the unique deep red lip that is arranged in a V-shaped formation on the flower, *C. lowianum* is used as a material for bonsai and is threatened by over-collection from its natural habitat for horticultural purposes.

The complete chloroplast genome sequences of *C. lowianum* was obtained (GenBank Accession No. MT576628). The genome sequences and features can be used to determine the phylogenetic relationship of *C. lowianum* and provide in-depth research into the chloroplast. In addition, it plays an essential role in understanding the diversity of *C. lowianum*. The specimens were collected from the Flower Research Institute of the College of Horticulture and Landscaping, Yunnan Agricultural University, Kunming, Yunnan Province, China (25°07′43″N, 102°44′54″E), and specimens were deposited in the Herbarium of Kunming Institute of Botany of CAS (specimen code: CY005). A modified CTAB method (Doyle and Doyle 1987) was used to extract the entire chloroplast DNA of *C. lowianum* from fresh mesophyll tissue.

Sequencing was performed using the Illumina NovaSeq conducted by GENOSEQ Technologies Limited Company (Wuhan, China). The raw reads and clean reads were obtained and then were assembled by SPAdes (Dierckxsens et al. 2017). The assembled contigs were compared with the chloroplast genomes of the closely related species through the use of blastn (version: BLAST 2.2.30+; parameter: -evalue 1e–5). Then the contigs were checked, selected, and adjusted to obtain the final data. The chloroplast genome was annotated and mapped using GeSeq (Tillich et al. 2017).

The length of the complete chloroplast genome of *C. lowianum* is 1,55,447 bp. The genome had a characteristic quadripartite circular structure that included one pair of inverted repeat regions (IRs, 26,710 bp), one large single-copy region (LSC, 84,184 bp), and one small single-copy region (SSC, 17,843 bp). Additionally, the complete genome contains 74 messenger RNA genes, 30 transfer RNA genes, and 4 ribosomal RNA genes. The overall GC content of the *C. lowianum* chloroplast genome is 36.77%. Moreover, the GC content of IR regions (43.09%) is higher than the LSC region (34.30%) and the SSC region (29.49%).

To study the phylogenetic relationship of *C. lowianum*, a phylogenetic tree was constructed by using 21 complete chloroplast genomes of *Cymbidium* species and three Orchidaceae species were selected as an outgroup. All the sequences were downloaded from NCBI GenBank and then aligned using the online program MAFFT version 7. MEGA v7.0 was used to build the maximum-likelihood phylogenetic tree with 1000 rapid bootstrap replicates (Kumar et al. 2016). The phylogenetic tree analysis indicated that *Cymbidium lowianum* was closely related to *Cymbidium erythraeum* (Figure 1).

**Figure 1.**
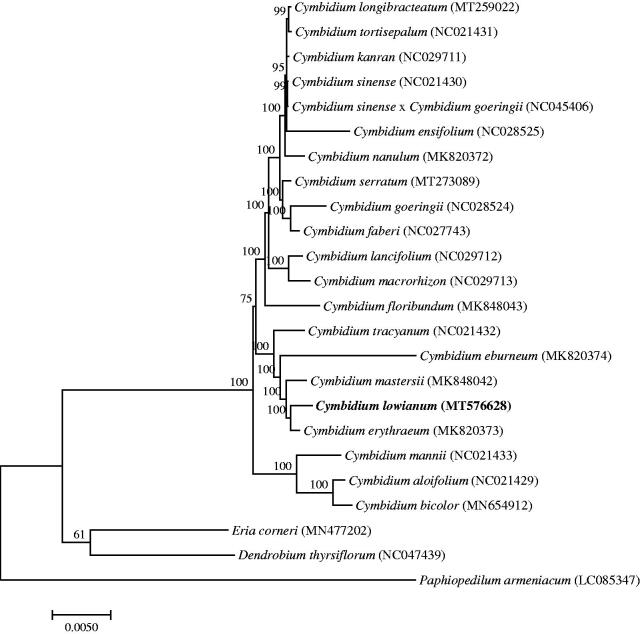
A Phylogenetic tree based on 24 complete chloroplast genome sequences of Orchidaceae species using the Maximum Likelihood (ML) analysis by MEGA v7.0. Bootstrap support values are indicated at each node.

## Data Availability

The data that support the findings of this study are openly available in GenBank of NCBI at https://www.ncbi.nlm.nih.gov, reference number MT576628.
